# Precise Measurements of the Quadratic Electro-Optic Effect in KH_2_PO_4_ Crystals Using a Sénarmont-Type System

**DOI:** 10.3390/ma14185435

**Published:** 2021-09-20

**Authors:** Marek Izdebski

**Affiliations:** Institute of Physics, Lodz University of Technology, Wólczańska 217/221, 93-005 Łódź, Poland; marek.izdebski@p.lodz.pl

**Keywords:** electro-optic effects, polarimetric method, Sénarmont configuration, KDP crystal

## Abstract

This paper presents precise measurements of the temperature dependencies of the quadratic electro-optic coefficients $g_{1111}-g_{1122}$ and $n_{e}^{3}g_{3333}-n_{o}^{3}g_{1133}$ in KH_2_PO_4_ crystals. In addition to traditional electro-optic coefficients describing changes in the function of an applied electric field, intrinsic coefficients, defined in terms of induced polarization, are also considered. Both intrinsic coefficients decrease with increases in temperature, but the relative temperature changes are of different orders of magnitude: 10^−4^ and 10^−3^ K^−1^. A Sénarmont-type setup was used for the electro-optic measurements. To achieve the best accuracy, a new approach was developed, in which, instead of using only one specific point on the modulator’s transmission characteristic, the operating point is changed during the measurements.

## 1. Introduction

Electro-optic coefficients are traditionally defined by expanding the components *B_ij_* of the relative optical dielectric impermeability tensor into a power series in the applied low-frequency electric field:

(1) $$B_{\mathrm{ij}}=\delta_{\mathrm{ij}}/n_{i}^{2}+r_{\mathrm{ijk}}E_{k}+g_{\mathrm{ijkl}}E_{k}E_{l}+\ldots ,$$

where δ*_ij_* is the Kronecker delta, *n_i_* are field-free refractive indices, and *r_ijk_* and *g_ijkl_* are the coefficients of the linear and quadratic electro-optic effects, respectively. The coefficients *r_ijk_* and *g_ijkl_* show a significant temperature dependence and vary widely in different materials. However, the changes in the *r_ijk_* coefficients are much smaller if we describe the impermeability tensor as a function of the induced polarization, rather than as a function of the applied field [1,2,3]. Due to the very small amount of experimental data available, it is currently unclear whether this rule can also be applied to the coefficients of the quadratic effect. Following the approach suggested by Pockels for the linear electro-optic effect [1,2], the intrinsic quadratic electro-optic coefficients *f_ijk_*, defined in terms of polarization, can be introduced as

(2) $$f_{\mathrm{ijkl}}=\frac{g_{\mathrm{ijkl}}}{\varepsilon_{0}^{2}\left(\varepsilon_{\mathrm{kk}}-1\right)\left(\varepsilon_{\mathrm{ll}}-1\right)}.$$

Similar coefficients may be defined using the Miller approach [2,3]:

(3) $$\delta_{\mathrm{ijkl}}=\frac{-\varepsilon_{\mathrm{ii}}\varepsilon_{\mathrm{jj}}g_{\mathrm{ijkl}}}{4\left(\varepsilon_{\mathrm{ii}}-1\right)\left(\varepsilon_{\mathrm{jj}}-1\right)\left(\varepsilon_{\mathrm{kk}}-1\right)\left(\varepsilon_{\mathrm{ll}}-1\right)},$$

where ε_0_ is the vacuum permittivity, ε*_ii_* and ε*_jj_* are the principal values of the optical dielectric tensor, and ε*_kk_* and ε*_ll_* are the principal values of the dielectric constant at a low modulating frequency.

The aim of this work was to experimentally investigate the temperature dependencies of the $g_{1111}-g_{1122}$ and $n_{e}^{3}g_{3333}-n_{o}^{3}g_{1133}$ quadratic electro-optic coefficients in KH_2_PO_4_ (KDP) crystals, and the corresponding intrinsic coefficients defined by Equations (2) and (3). The coefficients were chosen to show that it is possible, in the same material, to have one intrinsic coefficient with a clear temperature dependence and another with an almost constant value. Moreover, we show that, in the case of very weak temperature dependence, there is a significant difference between the coefficients defined by Equations (2) and (3). Although the properties of KDP crystals are quite well documented, the quadratic electro-optic coefficients, known from the literature, have only been measured at room temperature, or at low temperatures, near the paraelectric-ferroelectric phase transition. In this paper, we provide new data for temperatures from 25 °C to 85 °C for the $g_{1111}-g_{1122}$ coefficient and up to 90 °C for $n_{e}^{3}g_{3333}-n_{o}^{3}g_{1133}$.

This research required a method that would ensure the best accuracy of electro-optic measurements and allow the detection of very small temperature changes. Due to the limitations and disadvantages of existing methods, we developed a new method for measuring the coefficients of the quadratic electro-optic effect, which can also be used when the linear effect cannot be completely eliminated. Our method uses the well-known Sénarmont-type system, but in comparison to previous works, the following improvements have been made:

1. Our approach uses multiple operating points on the transmission characteristic of the modulator, instead of traditional measurement at only one specific point. The operating points are changed by a precise stepper motor that rotates the analyzer.
2. We propose a new advanced model of the experimental setup, which takes into account the possible differences in the transmission of the fast and slow waves in the sample and the quarter-wave plate, the inaccuracy in the phase difference introduced by the quarter-wave plate, the partial interference of two waves passing through the sample, and the apparent quadratic electro-optic effect that originates from the linear effect and nonlinear transmission characteristic of the modulator. The use of such a detailed model complicates the measurement procedure, but it seems necessary to achieve the highest accuracy in the electro-optic measurements.

## 2. Materials and Methods

Polarimetric methods for measuring electro-optic effects are based on the change in light intensity that occurs when the light passes through a sample, placed between two linear polarizers, under the influence of an applied electric field. The system may also include a retardation plate, placed in front of, or behind, the sample. In the most common configuration, the polarizers are crossed and oriented at an angle of ±45° relative to the planes of the fast waves in the sample and in the retardation plate. Assuming that there is no dichroism nor optical activity in the system, the intensity of the emerging light is given by

(4) $$\;I=\left(I_{\max}/2\right)\left[1-\cos\left(\Gamma \pm \gamma \right)\right],$$

where *I*_max_ is the maximum light intensity, Γ and γ are phase differences between the slow and fast waves in the sample and in the retardation plate, respectively, and the sign ± depends on the angle 0° or 90° between the azimuths of the fast waves.

In many materials submitted to electro-optic measurements, no significant natural birefringence is expected. For such materials, the configuration, described by Equation (4), is traditionally used with a quarter-wave plate (γ = 90°), which is added to obtain an almost linear relationship between the intensity of the transmitted light and small field-induced changes in Γ. This approach has been used in many electro-optic measurements performed for various uniaxial crystals, with a light beam along the optical axis, including LiNbO_3_, GaAS, KDP-type crystals, and others [4,5,6,7,8]. There are also known measurements of the Kerr effect in dispersions of nanoparticles [9] and in various liquids, such as mineral oils [10]. However, the method turns out to be sensitive to inaccuracies in the phase difference introduced by commercially available quarter-wave plates, as well as to additional birefringence that may appear both in the optical windows and in the sample itself.

In the case of a strongly birefringent sample, it is possible to use the temperature dependence of the linear birefringence to slowly move the working point along the *I*(Γ) characteristic [11,12,13,14,15]. In such a system, no additional retardation plate is needed (γ = 0°), and readings can be made at many points on the characteristic. This approach offers good sensitivity and accuracy, but it turns out to be very time-consuming. The method also causes some difficulties—for example, even small movements in the position of the crystal on its bases, during temperature changes, are observed as deviations of the actual *I*(*T*) dependence from the theoretical, strictly periodic, function.

The measurement system, without a retardation plate, was also used for samples that did not show natural birefringence, which corresponds to the minimum transmission point in Formula (4). Although the sensitivity of this method is low, it is sufficient when the quadratic electro-optic effect is very strong, such as for metal nanoparticles in glass [16,17].

A promising method that has been proposed recently, for determining electro-optic coefficients, uses a photoelastic modulator, operating as a dynamic retarder to compensate for the retardation introduced by the sample. The results so far reported for this method only include the linear electro-optic effect [18,19].

The electro-optic modulator may also have a configuration typical for the Sénarmont compensator. In this arrangement, the quarter-wave plate is placed behind the sample. The angle between the fast axis of the quarter-wave plate and the fast axis of the sample is ±45°, while the azimuth β of the analyzer can be freely adjusted. When the azimuth of the polarizer is +45°, the azimuth of the fast axis of the sample is 0°, and the azimuth of the quarter-wave plate is +45°, we obtain

(5) $$\;I=\left(I_{\max}/2\right)\left[1+\sin\left(2\beta +\Gamma \right)\right].$$

There are several known variations of measurement methods using the Sénarmont-type arrangement [4,5], but it is typically assumed that only one of the two specific operating points on the *I*(Γ) characteristic is used. The first option is to use the most linear part of the characteristic (5), which can be achieved by setting the analyzer at β = −Γ(0)/2, where Γ(0) is the field-free value of Γ. Since finding the maximum linearity point is neither easy nor accurate, the frequency-doubling electro-optic modulation (FDEOM) method uses the minimum transmission point. However, the FDEOM method provides relatively low sensitivity, which may be a significant limitation when the field-induced changes in Γ are very small.

The Sénarmont-type system is a common choice for many measurements of the linear electro-optic effect. In contrast, there are very few reports on measurements of the quadratic effect using maximum linearity conditions [20] or the FDEOM method [21,22]. To the best knowledge of the author, there are no reports in the literature on the separation of the quadratic electro-optic effect from the linear electro-optic effect and other unintended phenomena. However, slight inaccuracies in real systems mean that the linear effect often makes a dominant contribution to light modulation in configurations that theoretically exclude its occurrence. In such cases, it may not be clear whether the experimental results describe a true quadratic electro-optic effect or are a result of the linear effect.

In this paper, we show that the difficulties associated with the Sénarmont-type system can be overcome by changing the operating point during measurements. Since these changes can be achieved by rotating the analyzer, there is no need to exploit temperature changes, as in Refs. [11,12,13,14,15], which are more difficult to control. A more advanced and realistic mathematical model of the measurement system is also needed. Let us reconsider the Sénarmont-type system, but now, with the following assumptions:

**Assumption** **1.** *The phase difference γ introduced by the quarter-wave plate may differ from the ideal value of 90°.*

**Assumption** **2.** *The amplitude transmission coefficients through the quarter-wave plate Q_f_ and Q_s_ for the fast and slow waves, respectively, may not be equal.*

**Assumption** **3.** *The amplitude transmission coefficients through the sample S_f_ and S_s_ for fast and slow waves, respectively, may not be equal. The difference between the Q_f_ and Q_s_ coefficients or the S_f_ and S_s_ coefficients does not mean that the material used must show linear dichroism. In practice, this difference can also result from mechanical surface treatment, which may give precedence to one of the directions parallel to the surface. Another possible reason is that slightly different fractions of the fast and slow waves of light are reflected at the boundary between two media, as given by Fresnel’s equation.*

**Assumption** **4.** *The linear electro-optic effect need not be completely eliminated, and may make a significant nonlinear contribution to the dependence of the intensity of the emerging light on the applied electric field.*

**Assumption** **5.** *The fast and slow waves emerging from the sample may show only partial interference.*

Sénarmont-type optical systems may differ in the orientation of their components. In the studied configuration, the azimuth of the polarizer α = −45° or +45°, the azimuth of the fast axis of the sample ψ = 0° or 90°, and the azimuth of the fast axis of the quarter-wave plate θ = −45° or +45°. Assumptions 1–4 can be taken into account directly in the Jones matrix calculus. Using the general form of the Jones matrix, derived in Ref. [23], it can be found that the ratio of the emerging light intensity *I* to the intensity *I*_p_ behind the polarizer is given by

(6) $$\begin{array}{ll} I/I_{p} & =\frac{1}{8}\left(Q_{f}^{2}+Q_{s}^{2}\right)\left(S_{f}^{2}+S_{s}^{2}\right)\\ & +\frac{1}{4}\;\mathrm{pq}\left(Q_{f}^{2}-Q_{s}^{2}\right)S_{f}S_{s}\cos\Gamma \\ & +\frac{1}{8}\;q\sin\left(2\beta \right)\left(Q_{f}^{2}-Q_{s}^{2}\right)\left(S_{f}^{2}+S_{s}^{2}\right)\\ & +\frac{1}{4}\;s\cos\left(2\beta \right)Q_{f}Q_{s}\left(S_{f}^{2}-S_{s}^{2}\right)\cos\gamma \\ & +\frac{1}{4}\;p\sin\left(2\beta \right)\left(Q_{f}^{2}+Q_{s}^{2}\right)S_{f}S_{s}\cos\Gamma \\ & +\frac{1}{2}\;\mathrm{pqs}\cos\left(2\beta \right)Q_{f}Q_{s}S_{f}S_{s}\sin\gamma \sin\Gamma , \end{array}$$

where Γ and γ are the phase differences introduced by the sample and the quarter-wave plate, respectively, and *p*, *q*, *s* take values −1 or +1 depending on the azimuths α, θ, ψ. Namely: *p* = sgn(α), *q* = sgn(θ), and *s* = +1 for ψ = 0° or *s* = −1 for ψ = 90°.

The Jones calculus does not allow for partial interference of the two waves emerging from the sample. However, we can describe cases of total interference and no transmission of one of the two waves in the sample. Now, we will determine the effective intensity of the light emerging from the optical system (i.e., integrated over the entire cross-section of the beams), according to the approach proposed in Ref. [24] for double-refracted partially overlapping light beams:

(7) $$\;I=I_{f}+I_{s}+\left(I_{m}-I_{f}-I_{s}\right)D,$$

where *I*_f_ is the intensity in the zone in which only the fast wave propagates and *S*_s_ = 0, *I*_s_ is the intensity in the zone in which the slow wave propagates and *S*_f_ = 0, *I*_m_ is the intensity in the zone of full interference, in which neither of the two coefficients *S*_f_ or *S*_s_ is zeroed, and *D* ∈ [0, 1] is the relative overlap of the two beams. Substitution of Formula (6) into (7) yields

(8) $$\begin{array}{ll} I/I_{p} & =\frac{1}{8}\left(Q_{f}^{2}+Q_{s}^{2}\right)\left(S_{f}^{2}+S_{s}^{2}\right)\\ & +\frac{1}{4}\;\mathrm{pqD}\left(Q_{f}^{2}-Q_{s}^{2}\right)S_{f}S_{s}\cos\Gamma \\ & +\frac{1}{8}\;q\sin\left(2\beta \right)\left(Q_{f}^{2}-Q_{s}^{2}\right)\left(S_{f}^{2}+S_{s}^{2}\right)\\ & +\frac{1}{4}\;s\cos\left(2\beta \right)Q_{f}Q_{s}\left(S_{f}^{2}-S_{s}^{2}\right)\cos\gamma \\ & +\frac{1}{4}\;\mathrm{pD}\sin\left(2\beta \right)\left(Q_{f}^{2}+Q_{s}^{2}\right)S_{f}S_{s}\cos\Gamma \\ & +\frac{1}{2}\;\mathrm{pqsD}\cos\left(2\beta \right)Q_{f}Q_{s}S_{f}S_{s}\sin\gamma \sin\Gamma . \end{array}$$

In practice, measuring the light intensity behind the polarizer *I*_p_ could disturb the polarization of the light incident on the sample. To avoid this, we measure the intensity *I*_in_ of the light beam reflected by a beam-splitting mirror placed in front of the polarizer. It must be taken into account, however, that the light transmission through the polarizer *P* = *I*_in_/*I*_p_ may depend on its azimuth α due to possible deviations from the intended circular polarization of the incident light.

The transmission of light through the system *I*/*I*_in_ is a function of three azimuths, α, θ, and β, which can be changed easily during measurements. All of the terms in Formula (8) that contain the difference $Q_{f}^{2}-Q_{s}^{2}$ or $S_{f}^{2}-S_{s}^{2}$ can be eliminated by calculating the average light transmission $\bar{T}$ for the following two settings of *q* and β:

(9) $$\bar{T}\left(p,\;q,\;\beta \right)\overset{\mathrm{def}}{=}\frac{1}{2}\left[\frac{I\left(p,\;q,\;\beta \right)}{I_{\mathrm{in}}\left(p,\;q,\;\beta \right)}+\frac{I\left(p,-q,\;90{}^{\circ}-\beta \right)}{I_{\mathrm{in}}\left(p,-q,\;90{}^{\circ}-\beta \right)}\right].$$

Since the intensity of the laser light may change slightly during measurements involving various orientations of the optical elements, we consider *I*_in_ as a function of the orientations of all movable elements at the moment of measurement. Substitution of Formula (8) into (9) and the use of identities sin(*x*) = sin(180° − *x*) and cos(*x*) = −cos(180° − *x*) yields

(10) $$\begin{array}{ll} \bar{T}\left(p,\;q,\;\beta \right) & =\frac{1}{8}\;P\left(Q_{f}^{2}+Q_{s}^{2}\right)\left(S_{f}^{2}+S_{s}^{2}\right)\\ & +\frac{1}{4}\;\mathrm{pDP}\sin\left(2\beta \right)\left(Q_{f}^{2}+Q_{s}^{2}\right)S_{f}S_{s}\cos\Gamma \\ & +\frac{1}{2}\;\mathrm{pqsDP}\cos\left(2\beta \right)Q_{f}Q_{s}S_{f}S_{s}\sin\gamma \sin\Gamma . \end{array}$$

The total phase difference Γ introduced by the sample is the sum of the difference resulting from the natural birefringence Δ*n*_0_ and the changes induced by an applied electric field

(11) $$\Gamma =\frac{2\pi L}{\lambda }\left({\Delta n}_{0}+\mathrm{rE}+{\mathrm{gE}}^{2}\right),$$

where *L* is the length of the light path in the sample, λ is the wavelength of light, and *r* and *g* are the effective coefficients of the linear and quadratic electro-optic effects, respectively. In this work, we use a sinusoidal low-frequency modulating electric field

*E*(*t*) = *E*_0_ sin ω*t*. (12)

The average transmission $\bar{T}$ (10) can be decomposed into the sum of the DC component ${\bar{T}}_{0}$, the first harmonic ${\bar{T}}_{\omega }$, the second harmonic ${\bar{T}}_{2\omega }$, and other higher harmonics. The dependencies of ${\bar{T}}_{0},\;{\bar{T}}_{\omega }\mathrm{and}\;{\bar{T}}_{2\omega }$ on β can be treated as a sum of Fourier series, in which only the *a*_00_, *a*_02_, *b*_02_, *a*_12_, *b*_12_, *a*_22_, and *b*_22_ components can take non-zero values. In practice, the ratio *I*/*I*_in_ in Equation (9) is measured by photodetectors, which produce output voltages proportional to the respective light intensities *U*_in_~*I*_in_ and *U*~*I*. The voltage waveform *U*(*t*) can be resolved into the *U*_0_ component, measured by a DC voltmeter, and the *U*_ω_ and *U*_2ω_ components detected by a DSP lock-in amplifier at the reference frequency ω and its second harmonic. Using the measured voltages to describe the light intensities in Equation (9), we obtain

(13) $${\bar{u}}_{j\omega }\left(p,\;q,\;\beta \right)=\frac{1}{2}\left[\frac{U_{j\omega }\left(p,\;q,\;\beta \right)}{U_{\mathrm{in}}\left(p,\;q,\;\beta \right)}+\frac{U_{j\omega }\left(p,-q,\;90{}^{\circ}-\beta \right)}{U_{\mathrm{in}}\left(p,-q,\;90{}^{\circ}-\beta \right)}\right]=a_{j0}+a_{j2}\cos2\beta +b_{j2}\sin2\beta ,$$

where the values *j* = 0, 1 and 2 correspond to three different series. If the voltages are read for 2*n* azimuths β = *k*π/*n*, the coefficients in series (13) are given by

(14) $$a_{j0}=\frac{1}{2n}\overset{2n-1}{\underset{k=0}{\sum }}{\bar{u}}_{j\omega }\left(p,\;q,k\pi /n\right),$$

(15) $$a_{j2}=\frac{1}{n}\overset{2n-1}{\underset{k=0}{\sum }}{\bar{u}}_{j\omega }\left(p,\;q,k\pi /n\right)\cos\left(2k\pi /n\right),$$

(16) $$b_{j2}=\frac{1}{n}\overset{2n-1}{\underset{k=0}{\sum }}{\bar{u}}_{j\omega }\left(p,\;q,k\pi /n\right)\sin\left(2k\pi /n\right).$$

Following the convention typical for lock-in amplifiers, we assume that the readings show the RMS voltages |*U*_ω_| and |*U*_2ω_| and the phases ϕ_ω_ and ϕ_2ω_ ∈ (−180°; +180°], defined as

(17) $$\;U\left(t\right)=U_{0}+\sqrt{2}\left|U_{\omega }\right|\sin\left(\omega t+\phi_{\omega }\right)+\sqrt{2}\left|U_{2\omega }\right|\sin\left(2\omega t+\phi_{2\omega }\right)+\ldots$$

Due to the form of Equations (8), (11), and (12), the phase ϕ_ω_ can take only two values: 0° or 180°. The phase ϕ_2ω_ can also take only +90° or −90°. Therefore, both phases can be eliminated by considering the RMS voltages in Equations (13)–(16) as signed values

(18) $$\mathrm{sgn}\left(U_{\omega }\right)=\{\begin{array}{ll} +1, & \mathrm{for}\;\phi_{\omega }=0{}^{\circ},\\ -1, & \mathrm{for}\;\phi_{\omega }=180{}^{\circ}, \end{array}$$

(19) $$\mathrm{sgn}\left(U_{2\omega }\right)=\mathrm{sgn}\left(\phi_{2\omega }\right).$$

Knowing the experimental values of the coefficients *a*_02_, *b*_02_, *a*_22_, and *a*_22_, we are able to eliminate the terms *D*, *P*, *S*_f_, *S*_s_, and the contribution of the linear electro-optic effect described by the coefficient *r*. The transformations shown in Appendix A lead to the following formula for the effective coefficient of the quadratic electro-optic effect:

(20) $$\;g=-\mathrm{qs}\frac{\sqrt{2}\lambda }{{\pi \mathrm{LE}}_{0}^{2}}\frac{a_{22}b_{02}-b_{22}a_{02}}{a_{02}^{2}/C+b_{02}^{2}C},$$

where *C* is the calibration factor for the quarter-wave plate used in the measuring system. The factor takes the value 1 only for a perfect quarter-wave plate, or a value less than 1 for each imperfect element. The factor is given by

(21) $$\;C=\frac{2Q_{f}Q_{s}}{Q_{f}^{2}+Q_{s}^{2}}\sin\gamma =\frac{1-{\left(\Delta Q/Q\right)}^{2}}{1+{\left(\Delta Q/Q\right)}^{2}}\sin\gamma ,$$

where Δ*Q* = (*Q*_f_ − *Q*_s_)/2 and *Q* = (*Q*_f_ + *Q*_s_)/2. The value of the factor (21) must be known on the basis of other measurements made without a sample in the optical path. A review of methods for calibrating a quarter-wave plate is beyond the scope of this work.

We performed measurements of two KDP crystals for the following directions of the light and modulating field:

s = (0, 0, 1), E = (*E*, 0, 0), (22)

s = (1, 0, 0), E = (0, 0, *E*). (23)

Both crystals were cut in the form of right parallelepipeds with the dimensions 6.19 × 29.14 × 38.60 mm (*X* × *Y* × *Z*) for the crystal used in configuration (22) and 39.83 × 39.79 × 5.24 mm for configuration (23). In order to apply an electric field, two faces of each crystal were coated with silver conductive paint.

According to the matrices of linear and quadratic electro-optic tensors for $\bar{4}2m$ symmetry [6,25], the effective coefficient *r* = 0 for both configurations (22) and (23). The *g* coefficient in configuration (22) means

(24) $$\;g={0.5n}_{o}^{3}\left|g_{1111}-g_{1122}\right|,$$

and the sign of *g*_1111_ − *g*_1122_ can be determined from its relation to the azimuth of the fast wave in the sample. Assuming that the azimuth 0° is defined as the direction of the applied electric field, we obtain

(25) $$\;s=\mathrm{sgn}\left(g_{1111}-g_{1122}\right).$$

Formulas (20), (24), and (25) allow us to calculate the coefficient $n_{o}^{3}\left(g_{1111}-g_{1122}\right)$.

In the case of configuration (23),

(26) $$\;g=0.5\left(n_{e}^{3}g_{3333}-n_{o}^{3}g_{1133}\right),$$

and

(27) $$\;s=\mathrm{sgn}\left(n_{o}-n_{e}\right).$$

For *n*_o_ > *n*_e_, as in the case of KDP crystals, *s* = 1.

## 3. Experiment

**Figure 1:**
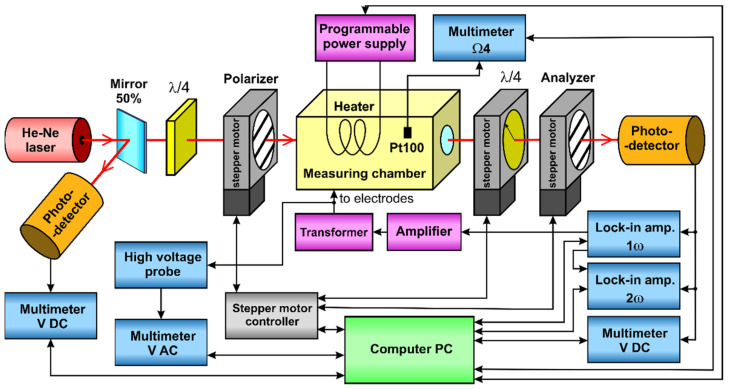
Block diagram of the measurement system.

The measurements were performed in the system shown in Figure 1. A Melles Griot 05-LHP-991 He-Ne laser was used as the light source, with a wavelength of λ = 632.8 nm. The light beam, reflected from a 50% beam-splitting mirror, was directed at a Thorlabs PDA36A-EC photodetector. The second light beam, which passed straight through the mirror, a quarter-wave plate, and an electro-optic modulator, was measured by a Thorlabs PDA100A-EC photodetector. An additional quarter-wave plate was placed in front of the polarizer to change the linear polarization, of the light emitted by the laser, to circular polarization. The electro-optic modulator consisted of a polarizer, the sample placed in the measuring chamber, a quarter-wave plate, and an analyzer. The orientations of the polarizer, quarter-wave plate, and analyzer were controlled by Thorlabs NR360S/M rotation stages, connected to a Thorlabs BSC203 three-channel controller. Two EG&G 7265 DSP lock-in amplifiers were used to split the voltage from the output photodetector into |*U*_ω_| and |*U*_2ω_| components and their phases ϕ_ω_ and ϕ_2ω_. One of the two amplifiers was also used as a modulating waveform generator. To obtain a modulating voltage of up to about 3000V RMS, a TELTO TSZ 90 VA high voltage transformer was used, driven by a Yamaha A-S501 amplifier. The *U*_in_ and *U*_0_ DC voltages, the modulating AC voltage, and the resistance of the temperature sensor were measured using four Keithley 2000 multimeters. As the modulating voltage exceeded the range of the multimeter, a Tektronix P6015A high voltage probe 1000:1 was used.

The measuring system included two serially produced multiorder quarter-wave plates made of quartz (Melles Griot, model 02WRQ001/632.8). The parameters of the plate behind the mirror can be omitted here, as they are irrelevant to the accuracy of the electro-optic measurements. With a quarter-wave plate placed between the sample and the analyzer, γ = 84.2693° and |Δ*Q*|/*Q* = 0.0157, which gives a calibration factor of *C* = 0.9945. It is worth noting that the deviation of *C* from the ideal value of 1 is mainly due to the inaccuracy of the phase difference γ. Such a large deviation from the value of 90° is not uncommon, and even greater inaccuracies have been reported in the case of commercially available quarter-wave plates (e.g., up to 10°, according to Ref. [26]).

Prior to the measurements, a selected crystal was placed in a 50 mm glass cuvette filled with methyl silicone oil with a viscosity of 50 cSt (OM50). The methyl silicone oil protected the hygroscopic crystal from moisture, reduced light reflection at the crystal faces, and improved heat transport. The cuvette was placed in the measuring chamber, which ensures a stable temperature with a maximum temperature error of ±0.2 °C and fluctuations of less than ±0.03 °C.

When the crystal, used in configuration (22), was placed on the bench, its optical axis was oriented along the laser beam with an accuracy of 0.05° by observing a conoscopic cross. As this method could not be used for configuration (23), the faces perpendicular to the *Y* and *Z* axes of the second crystal were oriented parallel to the light beam. Due to possible inaccuracies during cutting of the crystal, it can be expected that the actual orientations of the light and the applied field may differ from those intended in Equation (23) by up to ±1°.

The results of preliminary measurements, obtained for a single temperature of 25 °C, showed no significant dependence on the frequency of modulating voltage in the range from 217 to 1017 Hz (higher frequencies were not tested). A single frequency of 417 Hz was selected for further multi-temperature measurements. This value was chosen as a compromise between the noise level in the detection path and the load of the high voltage transformer, which show different frequency dependencies. It should also be noted that the selected frequency differs from all harmonics of the 50 Hz supply.

The measurements began at 25 °C, and the temperature was then increased by increments of 5 °C. The results presented in this paper are limited to maximum temperatures, at which we obtained stable readings, namely up to 85 °C for the crystal measured in configuration (22) and up to 90 °C in configuration (23). We observed a large increase in the electrical conductance of both KDP crystals at higher temperatures, which resulted in destabilization of the readings. This destabilization was due to the heat released in the crystals when subjected to high voltage.

The measurement procedure was as follows: after each temperature change, the computer program waited 10 h for the temperature measured in the oil to stabilize. The program then set all combinations of the analyzer azimuth from 0° to 355° in 5° steps, with two polarizer azimuths +45° and −45° and two quarter-wave plate azimuths +45° and −45°. After each change of the azimuths, the voltages *U*_in_, *U*_0_, *U*_ω_, *U*_2ω_, and the phases ϕ_ω_ and ϕ_2ω_ were measured for 16 constant levels of modulating voltage, increasing from about 750 to 3000 V RMS. The measurements were repeated 15 times for each modulating voltage, and the results were averaged. To apply Formula (20) to the results obtained at various voltage levels, we used the least squares method to fit the *a* coefficient in the relation $\left(a_{22}b_{02}-b_{22}a_{02}\right)/\left(a_{02}^{2}C^{-1}+b_{02}^{2}C\right)={a\;E}_{0}^{2}$.

The measurement procedure described above required about 24 h for each temperature level. This time-consuming procedure can certainly be accelerated, but in the present study, the priority was to achieve the highest accuracy.

## 4. Results and Discussion

**Figure 2:**
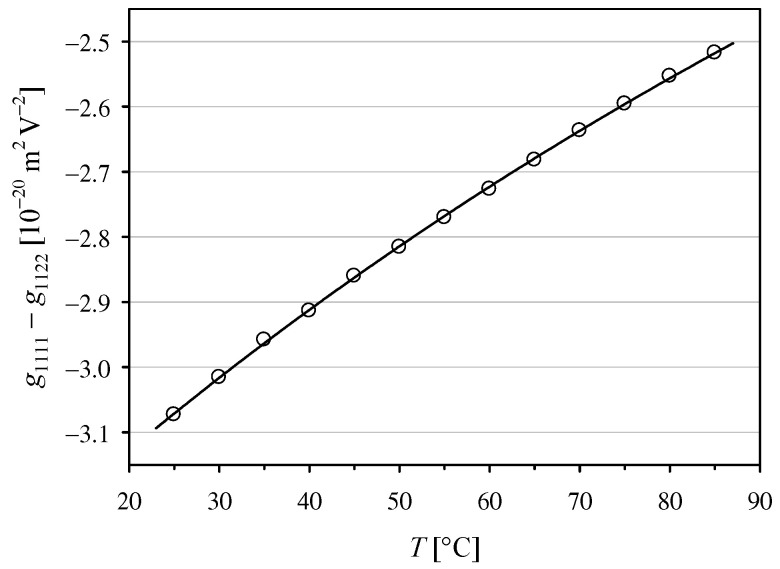
Temperature dependence of the quadratic electro-optic coefficient *g*_1111_ − *g*_1122_ with second-order polynomial interpolation *g*_1111_ − *g*_1122_ [10^−20^ m^2^ V^−2^] = −3.356 + 0.01215 *T* − 0.000027 *T*^2^ for *T* given in [°C].

The Formulas (20), (24), and (25) enable calculation of the coefficient $n_{o}^{3}\left(g_{1111}-g_{1122}\right)$. To find the values of *g*_1111_ − *g*_1122_, we used the temperature dependence of $n_{o}$ given by Ghosh and Bhar [27]. The averaged results obtained for the two polarizer azimuths −45° and +45° are shown in Figure 2. The value *g*_1111_ − *g*_1122_ = (−3.07 ± 0.03) × 10^−20^ m^2^V^−2^, found in this work for the KDP crystal at 25°C, and the 632.8 nm He-Ne laser is in good agreement with the previously reported values of *g*_1111_ − *g*_1122_ = (−3.1 ± 0.3) × 10^−20^ m^2^ V^−2^ [14] and |*g*_1111_ − *g*_1122_| = 2.5 × 10^−20^ m^2^V^−2^ [28] obtained by employing the polarimetric method, and with the values for the single coefficients *g*_1111_ = (−3.4 ± 0.5) × 10^−20^ m^2^V^−2^ and *g*_1122_ = (−0.2 ± 0.4) × 10^−20^ m^2^V^−2^, measured with an actively stabilized Michelson interferometer [29]. The temperature dependence obtained in this work cannot be compared with the results of previous studies due to the lack of data in the literature.

For optical frequencies $\varepsilon_{11}=n_{o}^{2}$, $\varepsilon_{33}=n_{e}^{2}$ and in Equation (3), we used the temperature dependencies of the ordinary $n_{o}$ and extraordinary $n_{e}$ refractive indices determined by Ghosh and Bhar [23]. The values of the low-frequency dielectric constants in Equations (2) and (3), for the paraeletric phase, can be expressed by a Curie-Weiss type formula:

(28) $$\varepsilon_{\mathrm{kk}}=\varepsilon_{\mathrm{kk}}^{\infty }+\frac{C_{\mathrm{kk}}}{T-T_{\mathrm{kk}}},$$

where, according to Deguchi and Nakamura, $\varepsilon_{11}^{\infty }=12$, $C_{11}=1.66{\;\times \;10}^{4}$ K, $T_{11}=-182$ K and $\varepsilon_{33}^{\infty }=7.00$, $C_{33}=2780$ K, $T_{33}=125.6$ K [30]. As the value of *T*_11_ seemed surprising, we verified that the values for ε_11_, calculated from Equation (28), were in very good agreement with our own measurements made with the LCR meter GW INSTEK LCR-6100.

**Figure 3:**
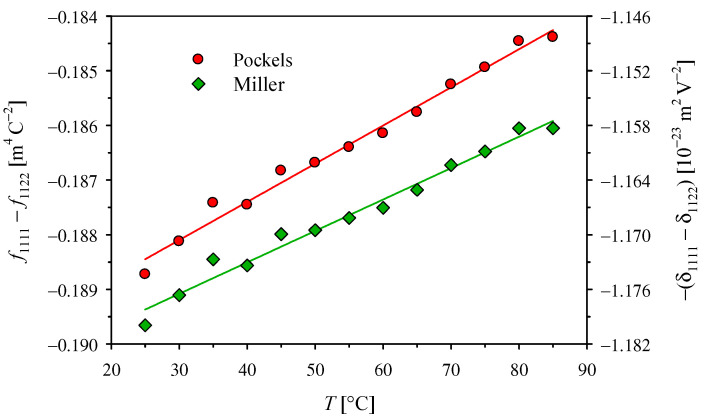
Temperature dependence of the intrinsic quadratic electro-optic coefficients with the linear interpolations *f*_1111_ − *f*_1122_ = *f*_0_(1 + *a*_f_ *T*) and *δ*_1111_ − *δ*_1122_ = δ_0_(1 + *a*_δ_ *T*), where the values at *T* = 0 °C are *f*_0_ = −0.19019(15) m^4^ C^−2^ and δ_0_ = 1.1868(10) × 10^−23^ m^2^V^−2^, and the temperature coefficients are *a*_f_ = −3.67(13) × 10^−4^ K^−1^ and *a*_δ_ = −2.91(13) × 10^−4^ K^−1^.

The absolute values of the intrinsic coefficients $f_{1111}-f_{1122}$ and $\delta_{1111}-\delta_{1122}$ presented in Figure 3 decrease, almost linearly, with the temperature increase. However, the changes are so small that we can notice a lower temperature coefficient of $\delta_{1111}-\delta_{1122}$, which results from the temperature dependence of ε_11_ at optical frequencies, included only in the Miller’s approach. Due to the lack of relevant data in the literature, we can only compare the value of −3.67 × 10^−4^ K^−1^ obtained here for the temperature dependence of the $f_{1111}-f_{1122}$ intrinsic coefficient in the KDP crystal with the value of −9.0 × 10^−4^ K^−1^ for ADP crystal [12]. It is worth noting that the use of the method described in [12] for KDP crystal would make it difficult to observe such a weak temperature dependence against the scatter of individual values.

**Figure 4:**
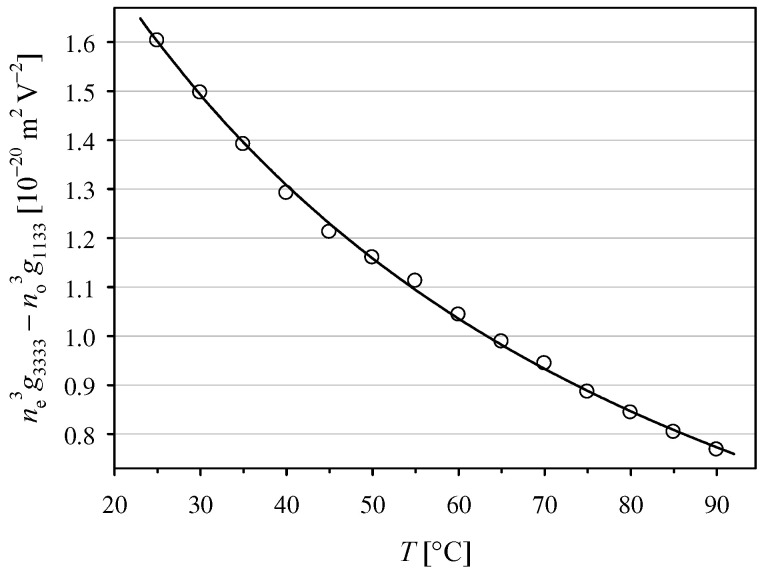
Temperature dependence of the quadratic electro-optic coefficient $n_{e}^{3}g_{3333}-n_{o}^{3}g_{1133}$ [10^−20^ m^2^ V^−2^] = 2.139 − 0.02492 *T* + 0.000109 *T*^2^ for *T* given in [°C].

Configuration (23) was used to perform measurements of the $n_{e}^{3}g_{3333}-n_{o}^{3}g_{1133}$ effective coefficient. The averaged results obtained for the two polarizer azimuths −45° and +45° are shown in Figure 4. To our knowledge, the temperature dependence of the effective coefficient $n_{e}^{3}g_{3333}-n_{o}^{3}g_{1133}$ in KDP crystal has not been reported previously. We can only compare our result $n_{e}^{3}g_{3333}-n_{o}^{3}g_{1133}$ = (+1.60 ± 0.04) × 10^−20^ m^2^ V^−2^ at 25 °C with the value $\left|n_{e}^{3}g_{3333}-n_{o}^{3}g_{1133}\right|$ = 3.1 × 10^−17^ m^2^ V^−2^ obtained using the static polarimetric technique [31], which means a difference of 3 orders of magnitude. However, many results obtained using the static method are known to differ enormously from newer data, as discussed in [29].

**Figure 5:**
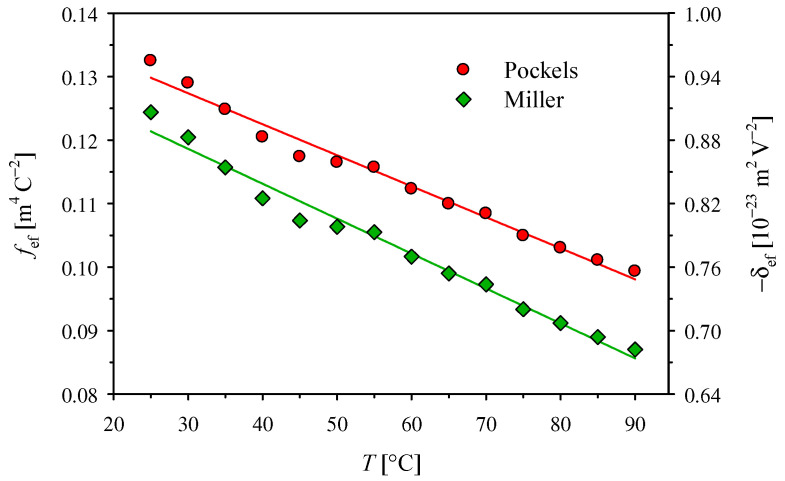
Temperature dependence of the intrinsic quadratic electro-optic coefficients $f_{\mathrm{ef}}=f_{3333}-n_{o}^{3}n_{e}^{-3}f_{1133}$ and $\delta_{\mathrm{ef}}=\delta_{3333}-n_{e}n_{o}^{-1}{\left(n_{o}^{2}-1\right)}^{2}{\left(n_{e}^{2}-1\right)}^{-2}\delta_{1133}$ with the linear interpolations *f*_ef_ = *f*_0_(1 + *a*_f_ *T*) and *δ*_ef_ = δ_0_(1 + *a*_δ_ *T*), where the values at *T* = 0 °C are *f*_0_ = 0.1420(12) m^4^ C^−2^ and δ_0_ = −0.9708(80) × 10^−23^ m^2^ V^−2^, and the temperature coefficients are *a*_f_ = −3.44(14) × 10^−3^ K^−1^ and *a*_δ_ = −3.40(14) × 10^−3^ K^−1^.

If we divide the coefficient $n_{e}^{3}g_{3333}-n_{o}^{3}g_{1133}$ by $\varepsilon_{0}^{2}{\left(\varepsilon_{33}-1\right)}^{2}$, we could arrive at $n_{e}^{3}{\;f}_{3333}-n_{o}^{3}{\;f}_{1133}$. However, it seems more advantageous to consider the temperature dependence of $f_{\mathrm{ef}}=f_{3333}-n_{o}^{3}n_{e}^{-3}f_{1133}$, where $n_{o}^{3}n_{e}^{-3}$ takes values close to 1 and changes only slightly from 1.0852 to 1.0835 in the temperature range from 25 to 90 °C [27]. Similarly, to reduce the impact of changes in the refractive indices, it is worth considering the effective intrinsic coefficient $\delta_{\mathrm{ef}}=\delta_{3333}-n_{e}n_{o}^{-1}{\left(n_{o}^{2}-1\right)}^{2}{\left(n_{e}^{2}-1\right)}^{-2}\delta_{1133}$. The absolute values of both coefficients, *f*_ef_ and δ_ef_, presented in Figure 5 decrease, almost linearly, with the temperature increase. However, the absolute values of the temperature coefficients here are 1 order of magnitude greater than those obtained for $f_{1111}-f_{1122}$ and $\delta_{1111}-\delta_{1122}$.

The scatter of individual results presented in Figure 4 and Figure 5 is clearly greater than those in Figure 2 and Figure 3. The two most important reasons are as follows:

(1) we do not have a method that would allow for such a precise orientation of the light beam along the *X* axis, as is possible in the case of the *Z* optical axis,
(2) in configuration (23), even slight changes in the crystal temperature lead to significant changes in the phase difference Γ, due to the temperature dependence of the natural birefringence Δ*n*_0_ and the length of the sample *L*.

**Figure 6:**
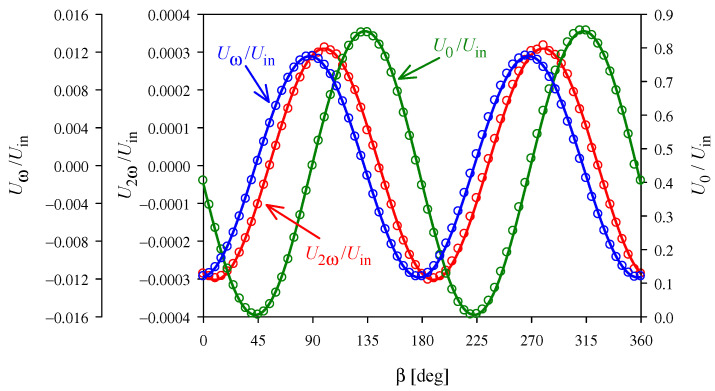
Example of experimental data obtained in the configuration (23) for *T* = 30°C, α = −45°, θ = −45°, and modulating voltage 3060 V RMS. The solid lines show interpolations of the type *U*_X_/*U*_in_ = *a* + *b* sin(2β + φ).

If the direction of the light differs slightly from that assumed in configurations (22) and (23), the linear effect is not completely eliminated. As the linear effect is much stronger than the quadratic effect, even small inaccuracies of 1° mean that the light modulation at the second harmonic of the modulating field must be measured against the background of the much stronger fundamental harmonic, as shown in Figure 6. Furthermore, both the linear and quadratic effects contribute to the modulation at the second harmonic, and the ratio of these contributions determines how precisely they can be separated.

Changes in the phase difference, due to unstable temperature, disturb the 180° period assumed in Section 2 for the dependence of *U*_0_, *U*_ω_, and *U*_2ω_ on β. Our experiments show that the accuracy of the temperature stabilization is limited, mainly, due to the heat generated in the crystal by the applied alternating voltage. The impact of this phenomenon on the accuracy of the measurements can be reduced by repeating a certain fixed cycle of the modulating voltage after each change of the temperature set in the thermostat. We start to collect data for calculation of the electro-optic coefficient only when the *U*_0_ change cycle stabilizes. As can be seen in Figure 6, the period of the dependencies obtained in this way does not differ significantly from 180°, even for the more difficult configuration (23).

Since the mathematical model of the measurement system proposed in this paper is relatively complex compared to previous works, it is worth considering whether the accuracy of the results justifies its use. Let us consider the substitutions γ = 90°, *Q* = *Q*_f_ = *Q*_s_ and *S* = *S*_f_ = *S*_s_, which simplify the Formula (8) to

(29) $$I/I_{p}=\frac{1}{2}\;Q^{2}S^{2}\left[1+\mathrm{pD}\sin\left(2\beta +\mathrm{qs}\Gamma \right)\right].$$

The form of Equation (29) shows that we no longer need to average the data as we did in Formula (13). Thus, the coefficients *a*_02_, *b*_02_, *a*_22_, *b*_22_ can be calculated from the Formulas (15) and (16) with the voltage ratios *U*_0_/*U*_in_ and *U*_2ω_/*U*_in_ substituted directly instead of ${\bar{u}}_{0}$ and ${\bar{u}}_{2\omega }$. The value of the effective electro-optic coefficient follows from Formula (20), but now the simplifications lead to *C* = 1. Using this simplified model, four values of *g* can be calculated for each temperature, which result from the data obtained for four combinations of polarizer and quarter-wave plate orientations. In the case of the more accurate model presented in Section 2, averaging the data obtained for the two quarter-wave azimuths in Formula (13) reduces the number of results to two for each temperature. The standard deviations σ_4_ and σ_2_, calculated from these values, are obviously unreliable. Therefore, we calculate the root mean square values σ_4RMS_ and σ_2RMS_ from the deviations σ_4_ and σ_2_, obtained for the individual temperatures. In the case of measurements of the $g_{1111}-g_{1122}$ coefficient, we obtain σ_4RMS_ = 0.116 × 10^−20^ m^2^ V^−2^ and σ_2RMS_ = 0.010 × 10^−20^ m^2^ V^−2^, and for $n_{e}^{3}g_{3333}-n_{o}^{3}g_{1133}$ we obtain σ_4RMS_ = 0.090 × 10^−20^ m^2^ V^−2^ and σ_2RMS_ = 0.010 × 10^−20^ m^2^ V^−2^. The σ_2RMS_/σ_4RMS_ ratio is in both cases much less than the value 2^−0.5^ ≈ 0.7 that would be expected if only doubling the number of data used to calculate one value of *g* was significant. The achieved improvement is, therefore, mostly the result of removing the assumptions γ = 90°, *Q*_f_ = *Q*_s_, and *S*_f_ = *S*_s_, which were widely used in previous studies.

The accuracy of the traditional measurement method, based on the Sénarmont system [4,5,20,21,22], is limited both by the use of a simplified mathematical model of the measurement system and by the measurement procedure, which uses only one specific operating point on the transmission characteristic. If this is the maximum linearity point, then the traditional method is highly sensitive. However, the procedure for finding this point is neither easy nor precise. Our measurements performed for configuration (22) show that the total measurement inaccuracy of the traditional method is about one order of magnitude higher than that achieved using the improved approach proposed in this paper. For example, the traditional method leads to the result *g*_1111_ − *g*_1122_ = (−3.0 ± 0.3) × 10^−20^ m^2^ V^−2^ for *T* = 25 °C, whereas the improved method gives (−3.07 ± 0.03) × 10^−20^ m^2^ V^−2^. In the case of the FDEOM method, the minimum transmission point can be found relatively accurately, but the sensitivity is insufficient to detect a weak quadratic electro-optic effect when the linear effect cannot be effectively eliminated.

## 5. Conclusions

We have measured the temperature dependencies of the quadratic electro-optic coefficients $g_{1111}-g_{1122}$ and $n_{e}^{3}g_{3333}-n_{o}^{3}g_{1133}$ in KDP crystals for temperatures above room temperature. To our knowledge, this is the first study of its type for KDP crystals at temperatures far above the paraelectric-ferroelectric phase transition temperature. The absolute values of the coefficients $g_{1111}-g_{1122}$ and $n_{e}^{3}g_{3333}-n_{o}^{3}g_{1133}$ decrease significantly with increasing temperature. These changes are due, mainly, to the temperature dependence of the dielectric constants at low frequencies. The changes are, therefore, much smaller when we use intrinsic electro-optic coefficients, defined in terms of induced electric polarization, instead of the traditional applied electric field. The temperature dependence observed for the field along the *X* crystallographic axis is so weak that there is a visible difference between the temperature coefficients −3.67 × 10^−4^ and −2.91 × 10^−4^ K^−1^, relating to the intrinsic coefficients $f_{1111}-f_{1122}$ and $\delta_{1111}-\delta_{1122}$ defined according to the Pockels and Miller approaches, respectively. The temperature coefficients, however, are one order of magnitude greater when the field is applied along the *Z* axis. These results suggest that the electron and lattice contribution to the quadratic electro-optic effect has different weights for the field applied along the *X* and *Z* axes.

The study of temperature dependences, of the intrinsic coefficients, required an accuracy that could not be achieved using methods described in the literature. To improve accuracy and reduce the scatter of results, we developed a method based on the Sénarmont configuration, where measurements are made at multiple operating points on the transmission characteristic of the modulator, instead of at only one specific point. A further improvement was achieved by using a more realistic model of the measurement system. The model proposed in this paper takes into account possible differences in the transmission of fast and slow waves in the sample, and in the quarter-wave plate, as well as inaccuracy in the phase difference introduced by the quarter-wave plate, partial interference between two waves passing through the sample, and the linear and quadratic electro-optic effects that can occur simultaneously. The approach proposed in this paper could be applied to study the bulk properties of many other electro-optic materials, including various monocrystals, materials containing nano-crystals, and liquids that do not exhibit high unstable birefringence.

## Data Availability

The data presented in this study are available on request from the corresponding author.

## Appendix A. Derivation of Equation (20)

The phase Γ resulting from Equations (11) and (12) can be decomposed into DC and AC components. To simplify further formulas, however, it is worth considering, not the Γ itself, but the DC and AC components of  qsΓ+(1−p)π/2:

(A1) $$\varphi =\left(1-p\right)\frac{\pi }{2}+\mathrm{qs}\frac{\pi L}{\lambda }\left({2\Delta n}_{0}+{\mathrm{gE}}_{0}^{2}\right),$$

(A2) $$\Delta \Gamma =\mathrm{qs}\frac{\pi L}{\lambda }\left({2\mathrm{rE}}_{0}\sin\omega t-{\mathrm{gE}}_{0}^{2}\cos2\omega t\right).$$

Equation (10) can now be rewritten as

(A3) $$\begin{array}{l} \bar{T}(p,q,\beta )\\ \;=\frac{1}{8}\;P\left(Q_{f}^{2}+Q_{s}^{2}\right)\left(S_{f}^{2}+S_{s}^{2}\right)\\ \;+\frac{1}{4}\;{\mathrm{DPS}}_{f}S_{s}\left[\left(Q_{f}^{2}+Q_{s}^{2}\right)\sin\left(2\beta \right)\cos\varphi +{2Q}_{f}Q_{s}\sin\gamma \cos\left(2\beta \right)\sin\varphi \right]\cos\Delta \Gamma \\ \;+\frac{1}{4}\;{\mathrm{DPS}}_{f}S_{s}\left[-\left(Q_{f}^{2}+Q_{s}^{2}\right)\sin\left(2\beta \right)\sin\varphi +{2Q}_{f}Q_{s}\sin\gamma \cos\left(2\beta \right)\cos\varphi \right]\sin\Delta \Gamma . \end{array}$$

As can be seen in Equation (A3), the Assumptions 1–3 made in Section 2 make it impossible to use the sum 2β + φ instead of individual values for 2β and φ. However, it seems useful to use the slightly different sums 2β + φ′ and 2β + φ″, which differ from 2β + φ due to the imperfections of the quarter-wave plate:

(A4) $$\begin{array}{ll} \bar{T}(p,q,\beta ) & =\frac{1}{8}\;P\left(Q_{f}^{2}+Q_{s}^{2}\right)\left(S_{f}^{2}+S_{s}^{2}\right)\\ & +\frac{1}{2}\;{\mathrm{DPS}}_{f}S_{s}\left[A^{\prime }\sin\left(2\beta +\varphi^{\prime }\right)\cos\Delta \Gamma +A^{\prime \prime }\cos\left(2\beta +\varphi^{\prime \prime }\right)\sin\Delta \Gamma \right], \end{array}$$

where

(A5) $$A^{\prime }=\frac{1}{2}\sqrt{{\left(Q_{f}^{2}+Q_{s}^{2}\right)}^{2}\cos^{2}\varphi +{\mathrm{4Q}}_{f}^{2}Q_{s}^{2}\sin^{2}\gamma \sin^{2}\varphi }$$

(A6) $$A^{\prime \prime }=\frac{1}{2}\sqrt{{\left(Q_{f}^{2}+Q_{s}^{2}\right)}^{2}\sin^{2}\varphi +{\mathrm{4Q}}_{f}^{2}Q_{s}^{2}\sin^{2}\gamma \cos^{2}\varphi },$$

(A7) $$\tan\varphi^{\prime }=\frac{{2Q}_{f}Q_{s}\sin\gamma }{Q_{f}^{2}+Q_{s}^{2}}\tan\varphi ,$$

(A8) $$\tan\varphi^{\prime \prime }=\frac{Q_{f}^{2}+Q_{s}^{2}}{{2Q}_{f}Q_{s}\sin\gamma }\tan\varphi .$$

Since the relationships (A7) and (A8) are ambiguous for φ, φ′, φ″ ∈ (−180°, +180°], it should be noted that the angles φ, φ′, φ″ lie in the same quadrant of the Cartesian coordinate system when sin γ > 0 (in practice γ takes values close to 90°). The expansions into power series cos ΔΓ ≈ 1 − ΔΓ^2^/2 + ΔΓ^4^/24 and sin ΔΓ ≈ ΔΓ − ΔΓ^3^/6 allow the average transmission $\bar{T}$ to be written as the sum of the DC component ${\bar{T}}_{0}$, the first harmonic ${\bar{T}}_{\omega }$, the second harmonic ${\bar{T}}_{2\omega }$, and higher harmonics, which will be omitted in further considerations. Neglecting very small terms, we find that

(A9) $${\bar{T}}_{0}\approx \frac{1}{8}\;P\left(Q_{f}^{2}+Q_{s}^{2}\right)\left(S_{f}^{2}+S_{s}^{2}\right)+\frac{1}{2}\;DPS_{f}S_{s}A^{\prime }\sin\left(2\beta +\varphi^{\prime }\right)(1-R^{2}),$$

(A10) $${\bar{T}}_{\omega }\approx {\mathrm{DPS}}_{f}S_{s}A^{\prime \prime }\cos\left(2\beta +\varphi^{\prime \prime }\right)R\left(1-R^{2}/2\right)\sin\omega t$$

(A11) $$\begin{array}{ll} {\bar{T}}_{2\omega } & \approx {\frac{1}{2}}_{\mathrm{DPS}f}S_{s}[A^{\prime }\sin\left(2\beta +\varphi^{\prime }\right)R^{2}\left(1-R^{2}/3\right)\\ & -A^{\prime \prime }\cos\left(2\beta +\varphi^{\prime \prime }\right)G\left(1-R^{2}\right)]\cos2\omega t, \end{array}$$

where

(A12) $$\;R=\mathrm{qs}\left(\pi L/\lambda \right){\mathrm{rE}}_{0},$$

(A13) $$\;G=\mathrm{qs}\left(\pi L/\lambda \right){\mathrm{gE}}_{0}^{2}.$$

As can be seen from Equation (A11), the modulation at the second harmonic has, as its sources, both the quadratic electro-optic effect and the square of the linear electro-optic effect. The latter contribution is extinguished for $\beta =-\varphi^{\prime }/2$ and $\beta =-\varphi^{\prime }/2+180{}^{\circ}$. In the former case, we obtain

(A14) $${\bar{T}}_{2\omega }(\beta =-\varphi^{\prime }/2)\approx -\frac{1}{2}{\mathrm{DPS}}_{f}S_{s}A^{\prime \prime }\cos\left(\varphi^{\prime \prime }-\varphi^{\prime }\right)G\left(1-R^{2}\right)\cos2\omega t.$$

The term *DPS*_f_ *S*_s_(1 − *R*^2^) can be eliminated using the magnitude of changes in the ${\bar{T}}_{0}$ component given by Equation (A9) for the variable azimuth β

(A15) $${\bar{T}}_{0,\max}-{\bar{T}}_{0,\min}\approx {\mathrm{DPS}}_{f}S_{s}A^{\prime \prime }\left(1-R^{2}\right).$$

Dividing the sides of Equation (A14) by (A15) we obtain

(A16) $$2\frac{{\bar{T}}_{2\omega }\left(\beta =-\varphi^{\prime }/2\right)}{{\bar{T}}_{0,\max}-{\bar{T}}_{0,\min}}\approx -\frac{A^{\prime \prime }}{A^{\prime }}\cos\left(\varphi^{\prime \prime }-\varphi^{\prime }\right)G\cos2\omega t.$$

The condition $\beta =-\varphi^{\prime }/2$ in Equation (A16) corresponds to the transition of the ${\bar{T}}_{0}$ component, given by Equation (A9) through its mean value when $d{\bar{T}}_{0}/d\beta >0$. In practice, however, a measurement method based on readings taken only in this particular situation does not seem to be the key to achieving the best accuracy. Moreover, the requirement for meeting the condition $\beta =-\varphi {}^{\prime }/2$ can be avoided by using a precise stepper motor to repeat readings for many β azimuths. This allows us to find the coefficients *a*_02_, *b*_02_, *a*_22_, and *b*_22_ in series (13). Comparison of Equation (A9) with the series (13) for *j* = 0 shows that the angle φ′ can be found as

(A17) $$\sin\varphi^{\prime }=a_{02}/{\left(a_{02}^{2}+b_{02}^{2}\right)}^{-1/2}\;\mathrm{and}\;\cos\varphi {}^{\prime }=b_{02}/{\left(a_{02}^{2}+b_{02}^{2}\right)}^{-1/2}.$$

Using the series (13) for *j* = 0 and *j* = 2 in the place of light transmissions, we can rewrite Equation (A16) in the form:

(A18) $$\sqrt{2}\frac{a_{22}b_{02}-b_{22}a_{02}}{a_{02}^{2}+b_{02}^{2}}=-\frac{A^{\prime \prime }}{A^{\prime }}\cos\left(\varphi^{\prime \prime }-\varphi^{\prime }\right)G.$$

Substitution of Equations (A5)–(A8), (A13), and (A17) into (A18) finally leads to Formula (20), which allows us to find the value of the effective coefficient *g* of the quadratic electro-optic effect.
